# Cardiac magnetic resonance biomarkers for cardiovascular complications in diabetes: a systematic review and meta-analysis

**DOI:** 10.3389/fcvm.2026.1817110

**Published:** 2026-05-28

**Authors:** Ning Li, Jitao Zhang, Yongyan Wang, Yugeng Li

**Affiliations:** 1 Department of Cardiovascular Medicine II, Qingdao Traditional Chinese Medicine Hospital, Qingdao Hiser Hospital Affiliated of Qingdao University, Qingdao, China; 2 Department of Cadre Health Care, Qingdao Traditional Chinese Medicine Hospital, Qingdao Hiser Hospital Affiliated of Qingdao University, Qingdao, China

**Keywords:** cardiac magnetic resonance, diabetes mellitus, extracellular volume, late gadolinium enhancement, meta-analysis, T1 mapping

## Abstract

**Background:**

Cardiovascular disease (CVD) is the leading cause of morbidity and mortality in diabetes mellitus (DM). Cardiac magnetic resonance (CMR) biomarkers, such as T1 mapping, extracellular volume fraction (ECV), and perfusion indices, have emerged as promising tools for early detection of cardiovascular complications in diabetes.

**Methods:**

We searched for original studies in PubMed, Scopus, Embase, ScienceDirect, and Google Scholar. Included studies enrolled patients with diabetes and were assessed using CMR biomarkers for diagnostic or prognostic outcomes. We extracted data related to study design, study population, CMR techniques, CMR biomarkers, and clinical outcomes. Random-effects meta-analyses were performed for outcomes reported in ≥2 studies.

**Results:**

Nineteen studies (*n* = 3,900+ patients) met inclusion criteria. CMR revealed subclinical myocardial changes in diabetic patients, including higher ECV, reduced strain, impaired perfusion, and increased prevalence of late gadolinium enhancement (LGE). Meta-analysis confirmed significantly elevated ECV (SMD = 0.838, *p* = 0.003), reduced myocardial perfusion reserve index (MPRI, SMD = 1.48, *p* = 0.033), and higher prevalence of LGE (SMD = 0.583, *p* < 0.001), while native T1 showed no significant differences, between-study heterogeneity was low across pooled analyses (overall I^2^ = 97.4%). The risk of bias was generally low to moderate.

**Conclusions:**

CMR biomarkers for diffuse fibrosis, focal scarring, and microvascular dysfunction provide important insights into myocardial involvement in diabetes and may detect subclinical abnormalities. Nevertheless, due to a small number of studies, the cross-sectional nature of most of the studies, and methodological diversity, these results should be viewed as preliminary. More large-scale, standardized, prospective trials are needed to confirm their contribution to early detection and risk stratification.

## 1 Introduction

Diabetes mellitus (DM) has become one of the most important public health issues, and the prevalence is growing at an epidemic pace (1–3). It is estimated that by 2050, 1 in 8 adults, will be living with diabetes, an increase rate of 46% (4). The burden of diabetes extends far beyond glycemic control, as chronic hyperglycemia initiates a cascade of metabolic and vascular disturbances that ultimately compromise multiple organ systems (5). DM has a number of important systemic effects. The most important cause of morbidity and mortality in diabetic patients is cardiovascular disease (CVD) (6). Patients with either type 1 or type 2 diabetes have been reported to have a two- to four-fold higher risk for heart failure, ischemic heart disease, arrhythmias and cardiomyopathy compared to non-diabetics and many of these complications present sub-clinically long before the onset of symptoms (7). It is therefore imperative that reliable, accurate and non-invasive methods be used as early as possible for diagnosing high-risk individuals.

The disease known as DM involves multiple concurrent metabolic processes along with vascular changes and inflammatory responses (8). Changes in glucose, lipid levels, and insulin activity in diabetes patients result in the impairment of endothelial function along with myocardial fibrosis and microvascular rarefaction while affecting energy processes (9). Functional changes in diabetic cardiovascular complications remain hidden until after structural changes which traditional assessments like echocardiography and ECG fail to detect. Researchers are increasingly focusing on advanced imaging technologies that map structural and functional changes as well as tissue alterations in hearts affected by diabetes.

Emerging technologies such as advanced signal processing, magnetic field generators, and super-resolution algorithms have expanded the potential of biomedical imaging and diagnostics (10). In this context, cardiac magnetic resonance imaging (CMRI) has been suggested as a possible complementary/alternative imaging modality, as it has the advantage of having no ionizing radiation and constant developments in hardware, pulse sequences, and motion compensation strategies (11). This imaging modality remains unique for its ability to provide a complete non-invasive assessment of cardiac anatomy and function during one examination. Conventional volumetric and functional imaging using CMR has been used for many years, but quantitative parametric mapping techniques, including native T1, T2, extracellular volume fraction (ECV), and T2* mapping, are rapidly advancing the capability of CMR to interrogate previously undetectable diffuse myocardial processes. Quantitative strain analysis, myocardial perfusion mapping, and fat-water separation techniques also provide further valuable tools (12).

Emerging data show the relationship between these CMR biomarkers and important cardiovascular outcomes in diabetes (13). Native T1 and ECV can be increased due to diffuse interstitial fibrosis that may be a phenotype of diabetic cardiomyopathy. T2 mapping can measure myocardial edema and inflammation. Impaired myocardial strain can indicate contractile dysfunction prior to a reduction in ejection fraction (14). Myocardial perfusion mapping provides an objective measure of myocardial blood flow that can be altered in microvascular dysfunction, another phenotype of diabetes that is common, but underrecognized.

However, the clinical role of CMR in diabetes has not been completely established yet. The results of individual studies in this field are quite heterogeneous. The differences in study population, sample size, CMR protocol and methodology, selected biomarkers, and study design may contribute to the non-uniformity of their conclusions (15). Research demonstrates that CMR markers provide superior prognostic value beyond traditional risk factors and echocardiography in some studies whereas other research finds these markers lack additional predictive utility (16). Technical variability across centers along with limited CMR availability and cost-effectiveness concerns restricts the broad application of CMR-derived biomarkers in standard clinical settings.

Since CMR techniques evolve quickly and CVD dominates diabetes-related mortality statistics, it becomes essential to perform a systematic literature review. The existing literature on diabetic cardiomyopathy, cardiac imaging in diabetes patients and myocardial tissue characterization has been summarized through narrative reviews (17). However, to our knowledge, no systematic review and meta-analysis of MRI biomarkers for cardiovascular complications in diabetes has been performed. It is essential to evaluate the diagnostic and prognostic capabilities of these biomarkers through systematic analysis while pinpointing current methodological shortcomings and setting research priorities.

Therefore, this systematic review and meta-analysis aims to thoroughly evaluate published research on CMR biomarkers for diabetic patients regarding diagnostic accuracy and prognostic and clinical usefulness across multiple cardiovascular outcomes such as heart failure, ischemia, fibrosis, and microvascular disease. The 10-year period was selected to include the recent work on CMR as well as the most recent clinical evidence. This review establishes a crucial foundation for further research and clinical application while directing the field toward its full potential in early detection and risk stratification which yields better patient outcomes for diabetes.

## 2 Methods

### 2.1 Literature search

The research team performed a thorough literature search across PubMed, Scopus, Embase, ScienceDirect, and Google Scholar to identify studies published from January 2015 to September 2025. We used a combination of MeSH terms and free-text words, including “diabetes mellitus,” “type 1 diabetes,” “type 2 diabetes,” “cardiac magnetic resonance,” “magnetic resonance imaging,” “cardiac MRI,” “MRI,” “T1 mapping,” “T2 mapping,” “extracellular volume,” “strain,” “perfusion,” and “cardiac biomarkers.” Boolean operators (AND, OR) were applied to refine the queries. The strategy was developed based on the PICO framework (Table 1), focusing on patients with diabetes (P), assessment by quantitative cardiac MRI biomarkers (I), comparison with healthy controls or conventional clinical/imaging assessments (C), and outcomes including diagnostic accuracy, prognostic performance, and cardiovascular complications such as fibrosis, ischemia, or cardiomyopathy (O). Reference lists of all included articles and relevant reviews were screened for additional eligible articles.

**Table 1:** PICO framework for search strategy.

Component	Description
P (Population)	Patients with diabetes mellitus (type 1 or type 2), both pediatric and adult populations
I (Intervention)	Cardiac MRI biomarkers (e.g., T1, T2, T2*, ECV, strain, perfusion mapping, myocardial fat fraction)
C (Comparison)	Healthy control groups, conventional cardiac imaging (e.g., echocardiography), or clinical risk factors
O (Outcomes)	Diagnostic accuracy, prognostic value, detection of cardiovascular complications (fibrosis, microvascular dysfunction, cardiomyopathy, heart failure, ischemia)

The full search strategies were prospectively archived in Supplementary Material S1.

### 2.2 Inclusion and exclusion criteria

We selected articles with original data that met the following criteria: Studies must include research on diabetic adults or pediatric patients and apply CMR biomarkers to assess cardiovascular issues while reporting diagnostic or prognostic results. Acceptable research methodologies for inclusion were randomized controlled trials, cohort studies, case-control studies, and cross-sectional studies. Our inclusion criteria permitted only publications written in English that were published from 2015 until 2025.

### 2.3 Study selection

All records were imported to EndNote (version X9) and duplicates removed. All article titles and abstracts were independently screened by two reviewers to determine eligibility before full-text articles underwent evaluation against set inclusion and exclusion criteria. When access to a full-text article was not possible, reviewers had to determine its eligibility based solely on available title and abstract information. In the case of disagreement, consensus was achieved with arbitration by a third reviewer, if needed.

### 2.4 Data extraction

A standardized data extraction form was developed in accordance with the aims of this review. Two reviewers independently extracted data from all eligible studies, and disagreements were resolved through discussion or consultation with a third reviewer. Extracted information was organized into the following categories (Table 2):

- Study identification: first author, year, country.
- Study design: cross-sectional, cohort, case-control, RCT.
- Population characteristics: sample size, age, diabetes type.
- Cardiovascular condition(s) assessed.
- CMR biomarkers: T1, T2, T2*, ECV, strain, perfusion, fat fraction, etc.
- CMR methodology: scanner strength, sequence type, contrast use, and analysis method.
- Main findings: statistically significant results and effect sizes.
- Conclusions/clinical implications.
- Notes/limitations reported by study author.

**Table 2:** Summary of key data and outcome measures.

First author, Year, Country	Study Design	Population	Age	DM Type	CV Condition(s)	CMR Biomarkers	CMR Methodology	Main Findings (statistical significance)	Conclusions/Clinical Implications	Limitations	Ref
Khdir *et al*., Egypt	Cross-sectional observationa	111 patients with CAD, 64 diabetics, 47 non-diabetics	Diabetics: 60.81 ± 8.56 yr; non-diabetics: 52.34 ± 13.34 yr	Not specified	CAD	EFV, PFV, LV EF, LV & RV volumes	1.5-T Philips Ingenia scanner; Cine SSFP for volumes; Fat volumes measured on short-axis slices using manual tracing	- Diabetics had higher EFV & PFV than non-diabetics (*p* < 0.05) - EFV ≥119.55 ml predicts high SS (AUC = 0.84, Sens 77%, Spec 82.5%) - PFV ≥125.05 ml predicts high SS (AUC = 0.83, Sens 75%, Spec 82.5%) - EFV & PFV correlated with HbA1c (*r* = 0.48) and SS (*r* = 0.71) - EFV & PFV negatively correlated with LV EF (*r* = −0.30, *r* = −0.27) - Intra-/Inter-observer ICC high (0.89–0.96)	Significant association between CMR-measured epicardial, and pericardial fat volumes and the complexity of CAD among diabetics as well as non-diabetics	Small sample; DM type not specified; single-center	(19)
Walters et al.*,* UK	Observational cross-sectional	84 T2DM with obesity, 36 healthy controls	T2DM: 50.5 ± 6.3; controls: 48.6 ± 6.2	T2DM	Subclinical cardiovascular dysfunction, stage B HF	Mean aortic distensibility, echocardiographic E/e'	1.5-T Siemens Aera; contrast-enhanced stress/rest perfusion CMR; analysis with cvi42 and Java Image Manipulation; CPET on bicycle ergometer	- VO₂ recovery associated with aortic distensibility (*β* = 0.218, *p* = 0.049). HR recovery associated with E/e' (*β* = −0.270, *p* = 0.024)	Alternative CPET indices (HR recovery, VO₂ recovery, VO₂VT) are impaired in T2DM and associate with subclinical cardiac dysfunction. These may help identify T2DM patients at elevated risk of progressing to symptomatic HF	Small cohort; more T2DM than controls; cross-sectional (no causality)	(20)
Shu et al., China	Prospective cohort	35 T2DM patients, 30 healthy controls	T2DM: 52 ± 12 yr; Controls: 49 ± 7 yr	T2DM	Cardiomyopathy, valvular disease, or LV hypertrophy	T1*ρ* mapping, native T1, ECV, GLS	1.5-T Philips Ingenia; 32-element body array coil; cine SSFP; native & postcontrast T1 mapping (MOLLI), T2 mapping (GraSE); T1*ρ* mapping with SL-prepared SSFP; analysis with Cvi42 & Matlab; 16 AHA segments; blinded observers	- ECV: 32.1 ± 3.2% (T2DM) vs. 26.2 ± 1.6% (controls), *P* < 0.001 - T1*ρ*: 51.6 ± 3.8 ms (T2DM) vs. 46.8 ± 2.0 ms, *P* < 0.001 - GLS: −16.5 ± 2.4% (T2DM) vs. −18.3 ± 2.6%, *P* = 0.015 - Native T1: no significant difference (*P* = 0.264) - Correlations: T1ρ & ECV *r* = 0.50; native T1 & ECV *r* = 0.25; T1ρ & HbA1c *r* = 0.52; ECV & HbA1c *r* = 0.61; ECV & diabetes duration *r* = 0.41	T1*ρ* is a promising non-contrast CMR technique for early detection of diffuse myocardial fibrosis in T2DM; may be more sensitive than native T1	Small sample size; single-center study; short-term evaluation; only T2DM patients without known CV disease included	(21)
Li et al., China	Prospective cohort	114 T2DM patients and 55 controls	T2DM: 63.37 ± 6.70 yr; Controls: 63.00 ± 5.97 yr	T2DM	MI, cardiomyopathy, valvular disease	- Stress PI - Global stress *Δ*T1 (T1 reactivity) - Global ECV - MPRI - Native/rest T1 value	- ECV: 31.79 ± 3.09% (T2DM) vs. 31.79 ± 3.09% (controls), *P* < 0.001 - 3.0-T Siemens MAGNETOM Trio Tim; 18-channel body coil+32-channel spine; ECG-triggered; adenosine infusion (140 μg/kg/min, 4 min); cine bSSFP, MOLLI T1 mapping (rest + stress), gadolinium contrast perfusion, LGE; perfusion images with IR-EPI; intra-/interobserver reliability excellent (ICC > 0.98)	- Stress PI lower in Q4 vs. Q2/Q3 (all *P* < 0.001) - MPRI between the T2DM and control groups (1.29 ± 0.18 vs. 1.68 ± 0.31; *P* < 0.001) - Stress *Δ*T1 impaired in T2DM vs. controls: 4.32 ± 1.64% vs. 6.17 ± 1.90% (*P* < 0.001) - Stress *Δ*T1 increased in Q2 (prediabetes, *P* = 0.018), decreased in Q3 & Q4 (*P* = 0.004, <0.001) - Global ECV increased in T2DM vs. controls: 31.79 ± 3.09% vs. 30.29 ± 2.18% (*P* < 0.001)	ECV rises early (even in prediabetes) and persists, suggesting diffuse fibrosis precedes perfusion decline; stress *Δ*T1 may be more sensitive than MPRI for detecting microvascular dysfunction. Early CMR markers can guide timely intervention.	Cross-sectional design prevents causal inference. No segmental MPRI analysis (focused only on global LV myocardium). CMR performed only at 3.0T	(22)
Boros et al., Brazil	Subanalysis of prospective MASS-V trial	155 stable CAD patients (67 with T2DM, 88 without)	T2DM: 62 ± 9; Controls: 62 ± 10 yr	T2DM	Multivessel CAD with preserved LVEF	GLS, circumferential strain (GCS), radial strain (GRS, post-contrast T1, ECV	1.5T CMR (cine SSFP, ShMOLLI T1 mapping, gadolinium contrast, LGE	T2DM patients had lower GLS, GCS, GRS (all *P* < 0.05) and higher ECV (25.7% vs. 23.5%, *P* < 0.01). Native and post-contrast T1 not significantly different. T2DM was an independent predictor of increased ECV and impaired strain after adjustment	Diabetes independently worsens myocardial strain and increases interstitial fibrosis (ECV) in CAD patients, even with preserved LVEF	Single-center, Variability in CMR protocols/magnetic field strengths. No T2 mapping. No diastolic dysfunction assessment. Prospective follow-up needed	(23)
Shen et al., China	Retrospective cohort	203 with T2DM without AR, 83 with T2DM + AR, 105 normal controls	T2DM without AR: 57 ± 11.5 yr; T2DM + AR: 65.1 ± 11.7 yr; Controls: 51.6 ± 10.6 yr	T2DM	Chronic aortic regurgitation in T2DM patients	LA phasic function indices (LAEF total, passive, active), LA longitudinal strain (*ε*s, εe, εa), LV strain (GLS), LV volumes, LV mass index, LVEF	3.0T; cine steady-state free precession; feature-tracking (cvi42) for strain; volumetric LA/LV analysis	T2DM patients (without aortic regurgitation) already had impaired LA function (lower LAEF, εs, εe) vs. controls. Presence of AR further aggravated LA and LV dysfunction, with severity-dependent declines in LA strain and LV function. Degree of AR was independently associated with εs and εa. LVEDV and LVMI also independently predicted LA strain impairment	AR synergistically worsens LA and LV dysfunction in T2DM patients. LA phasic strain is a sensitive marker of early myocardial dysfunction and atrioventricular interaction	Single-center retrospective study; no longitudinal follow-up; limited generalizability	(24)
Dattani et al., UK	Prospective cross-sectional	340 T2DM patients without CVD, 66 non-diabetic controls	T2DM: 64 (58–69); controls: 58 (54–65)	T2DM	Subclinical RV alterations, no HF or CAD	RV EDV, ESV, mass, GLS, GCS, longitudinal/circ	3.0 T Siemens (Skyra/Vida); bSSFP cine; stress perfusion (adenosine); T1 mapping	T2DM vs. controls: lower RV EDVi (84 vs. 100 mL/m, *p* < 0.001), higher RV GLS (26.3 vs. 23.5%, *p* < 0.001) and GCS (16.0 vs. 14.8%, *p* = 0.010),	Asymptomatic T2DM shows smaller RV volumes, hyperdynamic RV systolic strain but impaired RV relaxation. RV remodeling (lower EDV, higher cardiac output) independently determines reduced exercise capacity, surpassing LV parameters. Highlights need for right heart assessment in T2DM risk stratification	Age difference between groups (adjusted); relatively small control group; cross-sectional; RV volumes from LV short-axis stack (may miss outflow tract); no invasive pulmonary pressures	(25)
Lam et al., Singapore	Prospective cohort	148 asymptomatic T2DM patients	58.2 ± 8.5 yr	T2DM	Myocardial fibrosis	Myocardial fibrosis prevalence	High-resolution CMR (T1 mapping + LGE); Siemens scanner; concurrent serum biomarkers measured (MMP-2, MMP-9, galectin-3 via ELISA)	Myocardial fibrosis detected in 42% of asymptomatic T2DM patients. Biomarkers (MMP-2 *p* < 0.05, MMP-9 *p* = 0.01, galectin-3 *p* = 0.02) significantly elevated in fibrosis group. Strong correlation between CMR and biomarkers (*r* = 0.72, *p* < 0.001). Biomarker sensitivity 83%, specificity 77%	Combining high-resolution CMR with serum biomarkers provides a promising non-invasive strategy for early detection of myocardial fibrosis in asymptomatic T2DM. May enable earlier intervention to prevent progression to heart failure	Single-center study; no causality or longitudinal prediction. Findings need validation in multicenter, long-term cohorts	(26)
Bojer et al., Denmark	Cross-sectional	296 patients with T2DM and 25 non-diabetic controls	Not specified	T2DM	Myocardial interstitial fibrosis	ECV	1.5-T CMR; pre- and post-contrast T1 mapping (MOLLI); ECV calculated from native and post-contrast T1 values with haematocrit correction	Controls vs. T2DM without complications: similar ECV (27.4 ± 2.1% vs. 27.9 ± 2.6%, *P* = 0.4). T2DM with ≥1 complication vs. without: higher ECV (29.0 ± 3.3% vs. lower values, *P* = 0.02).	Patients with complications of diabetes show increased myocardial ECV (diffuse interstitial fibrosis), not seen in uncomplicated T2DM. Ischemic heart disease, autonomic neuropathy, and current (but not former) smoking are strongly and independently associated with higher ECV	No histological validation of fibrosis; single-center study. Cross-sectional design	(27)
Yeo *et al*., UK	Prospective cross-sectional	253 T2DM patients without CVD, 40 non-diabetic controls	T2DM: 63 ± 7; controls: 61 ± 8	T2DM	Subclinical diastolic dysfunction, no history of CVD or heart failure	Circumferential PEDSR, PLDSR, PEDSR:PLDSR ratio	Cardiac MRI (not fully specified) with bSSFP cine; feature tracking for diastolic strain rates	T2DM vs. controls: lower PEDSR (0.87 vs. 0.95 s^−1^, *p* = 0.043), higher PLDSR (0.80 vs. 0.67 s^−1^, *p* < 0.001), lower PEDSR:PLDSR ratio (1.18 vs. 1.53, *p* < 0.001).	Lower PEDSR:PLDSR in asymptomatic T2DM indicates impaired early diastolic relaxation and greater reliance on atrial contraction. This novel CMR feature tracking measure may assess global diastolic function, especially useful when echo windows are poor.	Cross-sectional design; no longitudinal follow-up	(28)
Bojer et al., Denmark	Cross-sectional observational study	296 patients with T2DM and 25 non-diabetic controls	Not specified	T2DM	Non-ischemic myocardial fibrosis; ischemic LGE also assessed	LGE, native and post-contrast T1 mapping, extracellular volume (ECV), MPRI, LV mass, LA volume	1.5T CMR; cine imaging, stress perfusion with adenosine, T1 mapping, and LGE imaging	- Patients with non-ischemic LGE lesions in comparison with patients without LGE lesions had increased myocardial mass (150 ± 34 vs. 133 ± 33 g, *P* = 0.02) - 9.5% of T2DM patients had non-ischemic LGE lesions - Patients with non-ischemic LGE had ↑ LV mass (*p* = 0.02), ↑ E/e′ (*p* = 0.04), ↑ LA volume (*p* = 0.049), ↑ NT-proBNP (*p* = 0.02), ↑ hs-troponin (*p* = 0.007), higher prevalence of retinopathy (48% vs. 25%, *p* = 0.009) and autonomic neuropathy (52% vs. 30.5%, *p* = 0.005) vs. those without lesions - MPRI lower in T2DM vs. controls (No quantitative value was reported), but no difference between non-ischemic LGE and no-LGE groups	A distinct non-ischemic LGE pattern was identified in ∼10% of T2DM patients, localized to the basal LV. These lesions were linked to higher LV mass, diastolic dysfunction, and microvascular complications	Excluded patients with advanced nephropathy, potentially missing associations with albuminuria; no myocardial biopsies performed; high-resolution LGE and feature tracking were not applied	(29)
Sørensen*et al*., Denmark	Cross-sectional	193 T2DM patients and 25 controls	T2DM: 59 ± 11 yr Controls: 57 ± 11 yr	T2DM	Coronary microvascular dysfunction	MPRI and LV	1.5-T Siemens Avanto; cine SSFP for volumes/function; adenosine stress (140 μg/kg/min) + rest perfusion (gadobutrol 0.075 mmol/kg)	- T2DM vs. controls: ↓ MPRI (3.0 ± 1.2 vs. 5.1 ± 1.5, *P* < 0.05), ↑ LV mass (138 ± 38 vs. 121 ± 25 g, *P* < 0.05), ↑ E/e* (7.8 ± 2.8 vs. 6.6 ± 1.5, *P* < 0.05). - Uncomplicated T2DM: ↓ MPR (3.8 ± 1.0 vs. 5.1 ± 1.5, *P* < 0.05) but similar LV mass/E/e* vs. controls.	Uncomplicated T2DM already shows reduced MPR (CMD) despite preserved LV mass and diastolic function; complications further impair perfusion and increase LV mass/diastolic dysfunction. Supports a direct link between CMD and diastolic dysfunction in T2DM; CMR MPR is a sensitive early marker	Perfusion analysis limited to mid-slice and excluded LGE segments; no histological validation	(30)
Gao et al., China	Prospective study	80 T2DM patients and 39 healthy controls	T2DM: 58.26 ± 11.65 yr Controls: 54.06 ± 13.19 yr	T2DM	Myocardial fibrosis	ECV; native T1; post-contrast T1; LV function	3.0T Siemens; Cine SSFP; MOLLI for T1 mapping; gadolinium 0.2 ml/kg; ECG gating; breath-hold; motion correction	The ECV value (all *p* < 0.001) was higher than both lower HbA1c group (36.23% vs. 32.19%, *p* < 0.001) and controls (36.23% vs. 29.73%, *p* < 0.001); HbA1c independent predictor of fibrosis (OR = 2.00, *p* = 0.014); ROC: HbA1c cutoff 7.1% predicted fibrosis (AUC = 0.79; sensitivity 66.7%; specificity 83.3%), native T1 in T2DM vs. controls (1,285.00 ± 54.09% vs. 1,279.83 ± 121.85)	T1 mapping is a sensitive biomarker of diffuse fibrosis in T2DM; HbA1c is an independent predictor useful for clinical decision-making and glucose control	Single-center; no myocardial biopsy	(31)
Ng et al., China	Prospective cohort	63 T2DM patients and 7 healthy controls	66 ± 4.4 yr	T2DM	High cardiovascular risk (Framingham ≥20%)	Global MPRI	Stress/rest CMR with 3T, cine imaging, first-pass perfusion	20.6% had obstructive CAD on CCA; 7.9% had silent infarcts; lower MPRI in DM vs. controls (1.43 ± 0.27 vs. 1.83 ± 0.31, *p* < 0.01)	1 in 5 high-risk asymptomatic DM patients have silent obstructive CAD; reduced MPRI may indicate microvascular dysfunction; stress CMR useful for screening	Small sample size; limited number of healthy controls; single-center; short follow-up	(32)
Storz et al., Germany	Prospective cohort	343 subjects without prior CVD, preserved LVEF; 218 controls, 78 prediabetes, 47 diabetes	56.1 ± 9.2 yr	Prediabetes and T2DM	No history of cardiovascular	LV function and morphology, LV mass, LGE, T1 mapping, ECV	3T; cine SSFP sequences; pre- and post-contrast T1 MOLLI 5 (3)3; LGE 10 min after gadolinium; LV remodelling index = LV mass/LV end- ECV calculated accounting for haematocrit	- LV remodelling index significantly higher in prediabetes (1.21 ± 0.27 g/mL) and diabetes (1.37 ± 0.38 g/mL) vs. controls (1.03 ± 0.24 g/mL, both *P* < 0.001) - ECV decreased in prediabetes (23.1 ± 2.4%) and diabetes (22.8 ± 3.0%) vs. controls (24.2 ± 2.8%, *P* = 0.007 and *P* = 0.003) - LGE prevalence higher in prediabetes (5%) and diabetes (7%) vs. controls (2%, *P* = 0.02 and 0.009)	Subjects with prediabetes and diabetes show early detectable myocardial changes, including higher LV remodelling index, increased cell volume, and mild LGE, even with preserved LVEF. Diffuse fibrosis appears less relevant at early stage. LV remodelling index may serve as a potential CMR biomarker for subclinical myocardial alterations	Relatively small sample size for ECV, cell volume, fibrosis measurements; post-contrast T1 mapping acquired at 10 min (guidelines recommend 15 min)	(33)
Liu et al.*,* China	Prospective cross-sectional	71 uncomplicated T2DM patients (31 newly diagnosed and 30 healthy controls)	T2DM: 53.99 ± 11.15; controls: 53.23 ± 8.59	T2DM	Subclinical myocardial dysfunction, no CAD or heart failure	PSSR, PDSR in radial	3.0 T Siemens; bSSFP cine; first-pass rest perfusion (gadobenate); LGE to exclude infarction; feature tracking (cvi42); 16-segment AHA model	Longitudinal PSSR (*β* = 0.243, *p* = 0.014), all three PDSR (*β* = 0.255–0.657, *p* ≤ 0.008), upslope (*β* = −0.399, *p* < 0.001), Max SI (*β* = −0.316, *p* < 0.001), but not TTM. Longitudinal PSSR correlated with upslope (*r* = −0.346, *p* = 0.003) and TTM (*r* = 0.515, *p* < 0.001)	3.0T contrast-enhanced CMR simultaneously detects subclinical myocardial dysfunction and impaired microvascular perfusion in early T2DM. PDSR is more sensitive than PSSR	Cross-sectional design	(34)
Markman et al.*,* USA	Prospective cohort	536 free of clinical CVD at baseline	64.1 ± 9.2 yr	T2DM	MI, angina, stroke, HF, atrial fibrillation	LA max volume index, LA min volume index, total LA emptying fraction	1.5 T scanners (GE, Siemens); tissue tracking CMR; biplane area-length method from 4- and 2-chamber cines	Incident CVD had larger LA max (32.1 vs. 26.8 mm^3^/m^2^) and min (19.4 vs. 14.2 mm^3^/m^2^) volumes, lower total (41.5 vs. 47.6%), passive (17.9 vs. 21.4%), and active (28.8 vs. 32.3%) EF (all *p* < 0.01)	CMR-measured LA min volume and LA emptying fractions predict incident CVD in asymptomatic diabetics, independent of traditional risk factors, LV mass, NT-proBNP, and max LA volume. LA function adds prognostic value beyond LA size	Diabetic-only cohort (cannot generalize to non-diabetics)	(35)
Heydari et al., USA	Retrospective cohort	173 patients with diabetes referred for suspected myocardial ischemia	61.7 ± 11.9 yr	T2DM	Investigated for myocardial ischemia	Stress perfusion defects (inducible ischemia); LGE (scar, infarct volume); LV function (LVEF, indexed LV volumes, regional wall motion abnormalities); inducible ischemia score (16-segment model)	3T Siemens; perfusion imaging (rest and stress); cine SSFP; LGE imaging 10–15 min after gadolinium (0.1 mmol/kg)	- Diabetics without inducible ischemia had low annual event rate (1.4%) vs. 8.2% in those with inducible ischemia (*P* = 0.0003) - LGE was lower in diabetics without inducible ischemia vs. inducible ischemia (3.0 ± 7.6 vs. 9.3 ± 12.6, *P* < 0.001) - Inducible ischemia strongest predictor of cardiac death/nonfatal MI (HR 4.86, *P* < 0.01; adjusted HR 4.28, *P* = 0.02)	Stress perfusion CMR provided robust independent prognostic value for diabetics with suspected ischemia. Stress perfusion CMR may be an effective tool for risk stratification and clinical decision-making in high-risk diabetic populations.	Single-center, relatively small sample size limited covariate adjustment in multivariable analyses. Potential referral bias as CMR results may have influenced downstream management	(36)
Levelt et al., UK	Prospective cohort	31 patients with T2DM and weight-matched healthy controls	Controls: 51 ± 9 T2DM: 55 ± 9 yr	T2DM	Investigated for coronary microvascular dysfunction	Rest and stress native T1 (maximal stress T1; MPRI; ECV; LV mass, LV concentricity, LV volumes, LVEF	3T Siemens; cine SSFP imaging; adenosine stress (140–210 μg/kg/min); ShMOLLI T1 mapping at rest and stress (non-contrast); first-pass perfusion with gadolinium (0.03 mmol/kg stress + rest, 0.15 mmol/kg total for perfusion + LGE)	- MPRI lower in T2DM vs. controls (1.60 ± 0.44 vs. 2.01 ± 0.42, *p* = 0.008) - Rest native T1 similar between groups (*p* = 0.59) - Stress T1 increased in both groups, but blunted in T2DM (*Δ*T1 = 4.1 ± 2.9% vs. 6.6 ± 2.6%, *p* = 0.007) - Maximal stress T1 lower in T2DM (1,244 ± 44 ms vs. 1,273 ± 44 ms, *p* = 0.045) - Negative correlations of stress T1 and *Δ*T1 with LV mass index and LV concentricity (*p* < 0.05) - No difference in LGE - No differences in LVEF, LV mass, or ECV	Patients with well-controlled T2DM, even without CAD, hypertension, or obesity, show blunted stress T1 response, consistent with coronary microvascular dysfunction and early LV concentric remodeling. Non-contrast T1 mapping during adenosine stress is a sensitive marker for subclinical microvascular dysfunction and may help detect early diabetic cardiomyopathy without gadolinium contrast	Small sample size, no long-term outcomes. Did not test inter-study reproducibility of stress T1 mapping. Cannot exclude contribution of obesity in some patients. Results limited to well-controlled T2DM, may not generalize	(37)

T2DM, type 2 diabetes; CAD, coronary artery disease; EFV, Epicardial Fat Volume; PFV, pericardial Fat Volume; ECV, extracellular volume fraction; GLS, global longitudinal strain; LGE, late gadolinium enhancement; MI, myocardial infarction; PI, perfusion index; IR-EPI, Inversion-recovery echo-planar imaging; SSFP, steady-state free precession; MOLLI, odified Look-Locker inversion recovery; LVEF, Left Ventricular Ejection Fraction; MPRI, myocardial perfusion reserve index.

### 2.5 Statistical analysis

For outcomes reported by at least two studies, a meta-analysis was conducted and presented in a forest plot. Standardized mean differences (SMD) and 95% confidence intervals (CI) were used for continuous variables. A random-effects model was used to estimate the pooled effect. Heterogeneity statistics (standard error, variance, relative weight) and the pooled SMD were extracted from the meta-analysis. Funnel plots were generated to provide a visual assessment of potential publication bias. The following outcomes were included in quantitative synthesis: ECV, myocardial perfusion reserve index (MPRI), native T1 relaxation time, and late gadolinium enhancement (LGE). Other outcomes with insufficient data were discussed narratively.

### 2.6 Risk of bias assessment

The methodological quality and risk of bias (ROB) were assessed using the ROBINS-I tool (18). Five domains were assessed: Researcher bias can occur from confounding factors and selection errors as well as missing data points and deviations from planned intervention protocols and through measurement errors during outcome assessment. The potential ROB for each domain was graded as low, moderate, serious, or critical. The overall ROB for each study was judged based on the most serious ROB in each domain. The assessment was independently performed by two reviewers, with disagreements resolved through consensus.

It is important to note that the ROBINS-I tool, though currently extensively used in non-randomized studies, might not be optimized to all of the covered study designs, especially cross-sectional and diagnostic biomarker studies. Thus, ROB evaluation needs to be considered carefully since some areas might not be able to represent methodological constraints in these types of studies.

## 3 Results

### 3.1 Study selection

**Figure 1:**
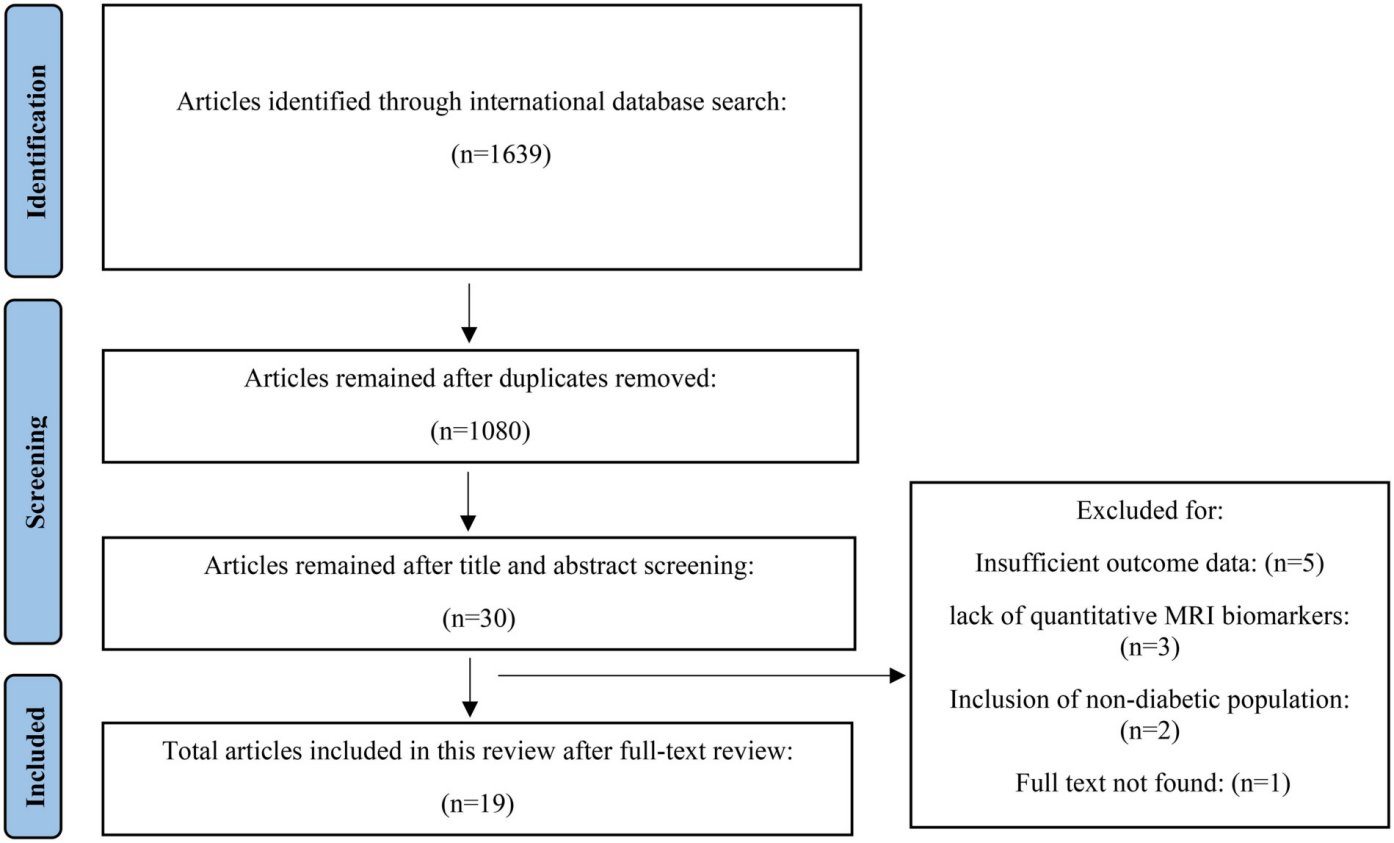
Flowchart of eligible studies included in this systematic review.

A total of 1639 records were identified through database searching. After removing 559 duplicates, 1,080 articles remained. Afterward, 1,050 of these records were excluded during title and abstract screening because they did not meet the eligibility criteria (i.e., not original articles, no relevant outcomes or no cardiovascular MRI data). We obtained and assessed 30 full texts of potentially relevant articles for eligibility. We excluded 11 articles after full-text review for reasons such as lack of MRI biomarkers, inclusion of non-diabetic population or no data on outcomes of interest. Finally, 19 original articles satisfied the inclusion criteria and were included in this systematic review. Figure 1 (PRISMA flow diagram) summarizes the process of study selection.

### 3.2 Key data and outcome measures of included studies

Nineteen studies published from 2016 through 2025 were identified. Studies were conducted in China, Egypt, Brazil, Denmark, Singapore, the United States, Germany, and the United Kingdom. The design of included studies was cross-sectional observational, prospective cohort, retrospective cohort, and subanalysis of randomized trials. The sample size of the included studies ranged from 31 to 536. Patients recruited had T2DM, prediabetes, and in some cases non-diabetic controls for comparison. The mean or median age of the patients included in the studies was typically between the mid-50s to the early 60s. Most of the included studies were examining adult T2DM patients, however one also included those with prediabetes. The investigation covered a variety of cardiovascular conditions such as coronary artery disease (CAD), myocardial ischemia (MI), subclinical diastolic dysfunction, angina, stroke, atrial fibrillation, cardiomyopathy, myocardial fibrosis, coronary microvascular dysfunction, high cardiovascular risk in asymptomatic patients and valvular disease including chronic aortic regurgitation.

A wide range of CMR biomarkers were assessed across the 19 studies, which included: native and post-contrast T1 mapping, T1*ρ* mapping, T2 mapping, ECV, MPRI, global and segmental strain parameters (GLS, GCS, GRS), stress perfusion indices, end-diastolic volume (EDV), end-systolic volume (ESV), peak systolic strain rate (PSSR), peak diastolic strain rate (PDSR), LA max volume index, LA min volume index, epicardial and pericardial fat volumes and LGE. Several studies also included stress CMR protocols with adenosine infusion to evaluate myocardial perfusion and stress T1 reactivity. Different methodologies were used, with both 1.5 Tesla and 3.0 Tesla scanners being utilized in the included studies, using cine steady-state free precession imaging, parametric mapping approaches (MOLLI, ShMOLLI, GraSE), and contrast-enhanced perfusion or LGE imaging. Commercial or in-house software was used for image analysis, with some studies providing information about intra- and interobserver reproducibility.

Primary endpoints across studies reported significant changes in CMR biomarkers in patients with diabetes when compared to non-diabetic controls. These biomarkers included higher ECV, reduced myocardial strain, decreased perfusion reserve indices, blunted stress T1 responses, and more frequent LGE in asymptomatic or well-controlled subjects with diabetes. Associations between CMR parameters and HbA1c, diabetes duration, or cardiovascular risk scores were shown in some studies. Clinical implications described in the articles showed the potential use of CMR-derived biomarkers for the identification of subclinical myocardial dysfunction, cardiovascular risk stratification and the early detection of pathological processes before the development of clear clinical disease. Markman et al. (35) demonstrated that left atrial minimum volume and emptying fractions independently predicted incident cardiovascular disease; Liu et al. (34) showed that peak diastolic strain rate was more sensitive than systolic strain in detecting early dysfunction; Yeo et al. (28) introduced the PEDSR:PLDSR ratio as a novel marker of diastolic impairment; Dattani et al. (25) found that right ventricular remodeling, not left ventricular parameters, determined reduced exercise capacity; and Walters et al. (20) linked alternative cardiopulmonary exercise testing indices to subclinical cardiac dysfunction. The main limitations were small sample sizes, single center design, cross sectional studies without longitudinal follow-up, heterogeneous protocols, and lack of histological validation.

### 3.3 Meta-analysis results

**Figure 2:**
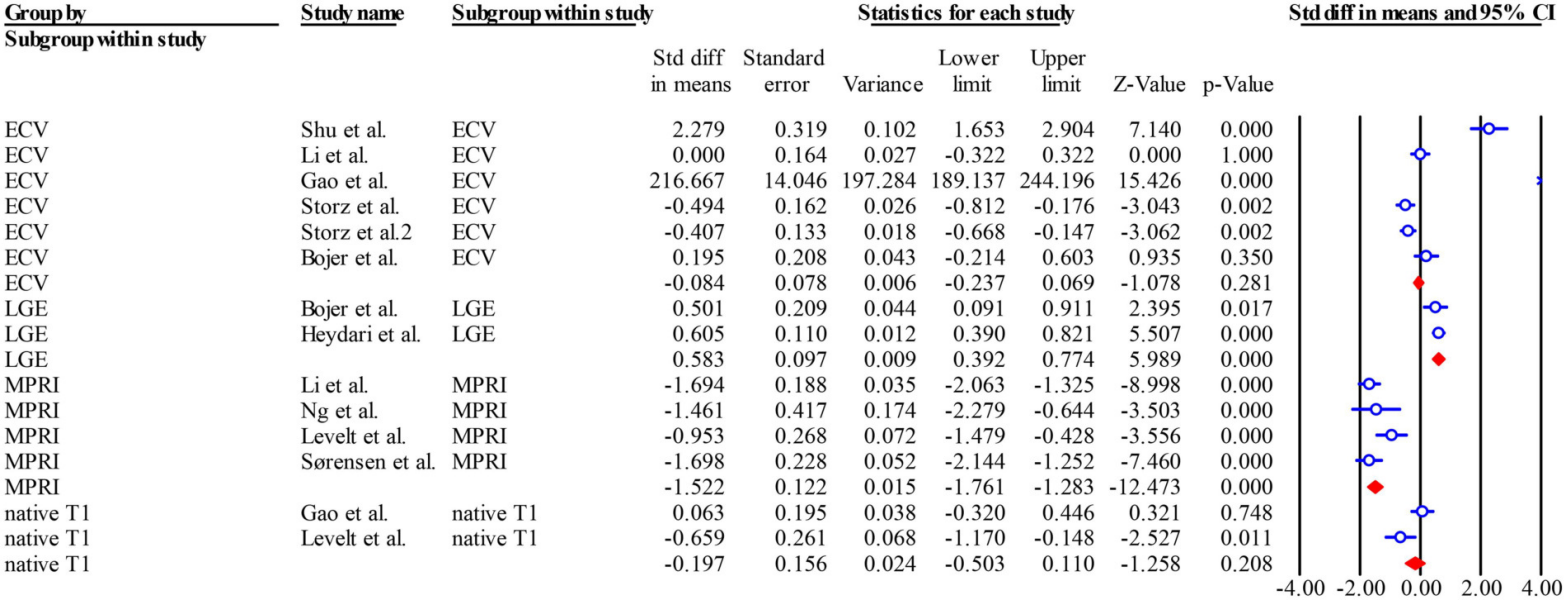
Forest plots of pooled outcomes. Forest plots showing the meta-analysis of extracellular volume (ECV), late gadolinium enhancement (LGE), myocardial perfusion reserve index (MPRI), and native T1 relaxation time in patients with diabetes compared with controls.

Six studies (21, 22, 27, 29, 31, 33) contributed to the pooled analysis of ECV, demonstrating a significant increase in diabetic patients (SMD = 0.838, 95% CI 0.295–1.488, *p* = 0.003), with high heterogeneity across studies (I^2^ = 98.3%). Two studies (29, 36) evaluated late LGE, with the pooled estimate indicating a significant association and higher prevalence among diabetes cases (SMD = 0.583, 95% CI 0.392–0.774, *p* < 0.001), with no observed heterogeneity between studies (I^2^ = 0%). Four studies (22, 30, 32, 37) examined MPRI, yielding a significant pooled effect (SMD = 1.48, 95% CI 1.127–1.838, *p* = 0.033), with moderate between-study heterogeneity (I^2^ = 49.6%). In contrast, two studies [31, 337] assessing native T1 relaxation time showed no significant difference (SMD = 0.307, 95% CI −0.336 to 0.951, *p* = 0.349), with no detectable heterogeneity (I^2^ = 0%). Overall, heterogeneity across pooled analyses was high (I^2^ = 97.4%).‏ Such heterogeneity indicates high levels of clinical and methodological variability, such as variations in the type of MRI sequences, strength of the scanners used, reference standards, and units of analysis. These findings are summarized in the corresponding forest plots (Figure 2).

**Figure 3:**
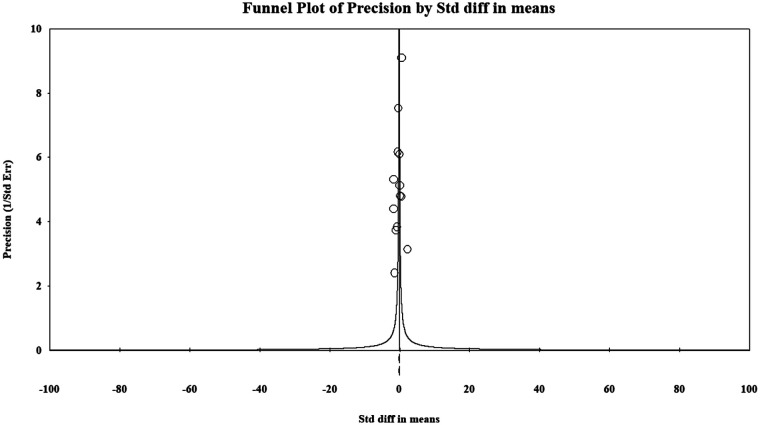
Funnel plots of pooled outcomes, illustrating the distribution of studies and visual assessment of potential publication bias.

Funnel plots were constructed for the outcomes included in the quantitative synthesis to evaluate the potential for publication bias (Figure 3). The plots provide a visual assessment of study distribution around the pooled effect size. For ECV, MPRI, and native T1, the limited number of contributing studies (two to six per outcome) restricts the interpretability of asymmetry patterns. The LGE analysis was also based on only two studies, which does not allow meaningful conclusions to be drawn from funnel plot inspection. Overall, while funnel plots were generated, the small number of studies per outcome precludes definitive assessment of publication bias.

### 3.4 Risk of bias assessment

**Figure 4:**
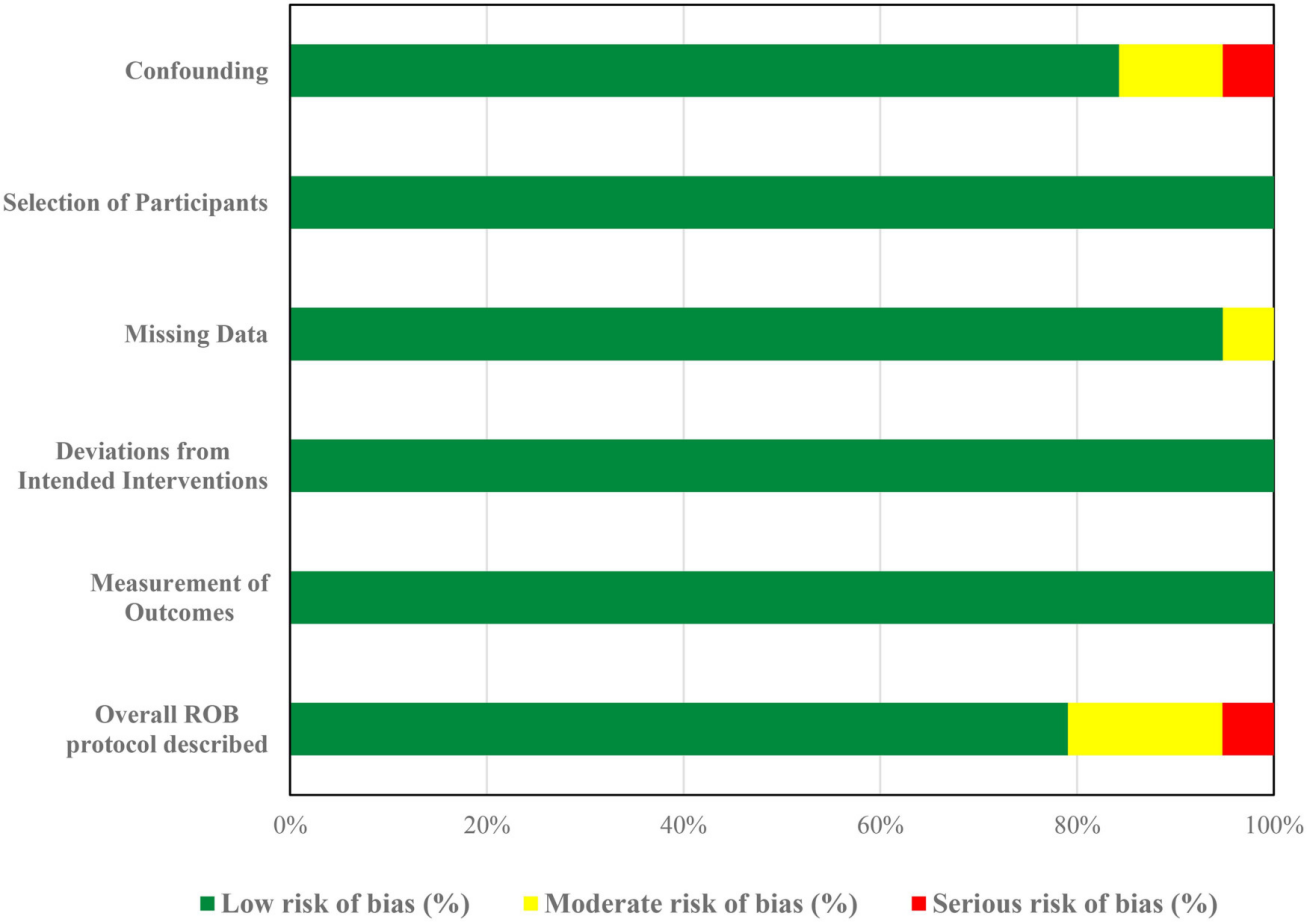
Distribution of ROB domains across all included studies.

Overall, the majority of the studies were judged to be at low or moderate ROB (Table 3, Figure 4). The most common source of potential bias was from confounding, mainly as a result of generalizability, retrospective design, and referral bias. Selection of participants was generally at low risk since most studies had clearly stated inclusion and exclusion criteria. Missing data were rarely reported and were generally not considered to affect the primary outcomes of interest, though in one study ECV measurements were only performed in a subset of the study population, not across the entire cohort, resulting in moderate ratings. Deviation from intended interventions were considered low across all studies, as the majority of studies used well-established and standard imaging protocols. ROB in measurement of outcome was also consistently low, as CMR endpoints were assessed using standard approaches in all studies. Overall, three studies had a moderate ROB, one study was rated as having a serious ROB, and the remaining studies were judged to have a low ROB. No studies were considered to be at critical ROB and thus excluded from the synthesis.

**Table 3:** Risk of bias assessment.

Study	Confounding	Selection of Participants	Missing Data	Deviations from Intended Interventions	Measurement of Outcomes	Overall ROB
Khdir et al.	Low	Low	Low	Low	Low	Low
Walters et al.	Low	Low	Low	Low	Low	Low
Shu et al.	Low	Low	Low	Low	Low	Low
Li et al.	Low	Low	Low	Low	Low	Low
Boros et al.	Moderate: CAD cohort limits generalizability	Low	Low	Low	Low	Moderate
Shen et al.	Moderate: retrospective design	Low	Low	Low	Low	Moderate
Dattani et al.	Low	Low	Low	Low	Low	Low
Lam et al.	Low	Low	Low	Low	Low	Low
Bojer *et al*. (27)	Low	Low	Low	Low	Low	Low
Yeo et al.	Low	Low	Low	Low	Low	Low
Bojer *et al*. (29)	Low	Low	Low	Low	Low	Low
Sørensen *et al*.	Low	Low	Low	Low	Low	Low
Gao et al.	Low	Low	Low	Low	Low	Low
Ng et al.	Low	Low	Low	Low	Low	Low
Storz et al.	Low	Low	Moderate: ECV only in subset	Low	Low	Moderate
Liu et al.	Low	Low	Low	Low	Low	Low
Markman et al.	Low	Low	Low	Low	Low	Low
Heydari et al.	Serious: referral bias (symptomatic cohort)	Low	Low	Low	Low	Serious
**Levelt et al.**	Low	Low	Low	Low	Low	Low

## 4 Discussion

CVD is the primary cause of morbidity and mortality in diabetic patients and affected individuals have a two- to four-fold increased risk compared with their non-diabetic counterparts (38–40). Even with advances in treatment, detecting subtle or early changes in the heart before symptoms appear remains a significant challenge (41). CMR imaging has gained attention as a powerful, non-invasive approach that can reliably capture structural, functional, and tissue-level changes in the myocardium (42). Biomarkers such as MPRI, native T1, ECV, and LGE provide detailed information about a number of key processes, such as ischemia, microvascular dysfunction, diffuse fibrosis and scarring that play an integral role in the pathogenesis and progression of diabetic heart disease (43, 44). This systematic review and meta-analysis provide a collated summary of current evidence of the clinical application of CMR in diabetes over the last 10 years.

### 4.1 Interpretation of main findings

Nineteen original studies were included in this review. All these demonstrate that CMR is able to identify subclinical myocardial changes in T2DM, despite normal ejection fraction. The most important CMR biomarkers were ECV, LGE, MPRI/ MBF, and strain parameters.

Several studies have shown augmented diffuse myocardial fibrosis in T2DM (45, 46). The reported significant higher ECV in diabetic patients by Shu et al., Li et al., Boros et al., and Gao et al. was related to HbA1c and duration of diabetes (21–23, 31). Bojer et al. also indicated that ECV did not differ between controls and uncomplicated T2DM but was significantly increased in patients with complications (29.0 ± 3.3%, *P* = 0.02) (27). Ischemic heart disease, autonomic neuropathy, and active smoking were all found to be separately predictive of higher ECV with the strongest effect observed with active smoking (2.0, 95% CI 0.73.3).

Focal scarring was evident in increased LGE prevalence. Bojer et al. also found non-ischemic mid-wall LGE lesions in approximately 9.5% of T2DM patients, which were linked to diastolic failure and microvascular issues (29). Meta-analysis affirmed increased LGE in diabetes (SMD = 0.583, *p* < 0.001).

Microvascular dysfunction was consistently detected. Levelt et al., Ng et al., and Li et al. showed reduced MPRI and blunted stress *Δ*T1 in T2DM (22, 32, 37). Sørensen et al. demonstrated that reduced myocardial perfusion reserve resulted from both elevated resting MBF and decreased stress MBF, with greater impairment in patients with albuminuria, retinopathy, or autonomic neuropathy (30). Lower stress MBF correlated with higher ECV (*R*^2^ = 0.37, *P* < 0.001) and diastolic dysfunction. Subclinical functional changes, including reduced global longitudinal strain and left atrial impairment, were reported by Boros et al. and Shen et al. Heydari et al. highlighted the strong prognostic value of stress perfusion CMR (23, 24, 36).

In these 19 studies, we now know that patients with diabetes exhibit a distinct pattern of subclinical myocardial changes detectable by CMR even when LVEF is preserved. The myocardium shows concentric remodeling with increased left LV and a higher remodeling index, accompanied by reduced MPRI indicating early microvascular dysfunction (30, 37). Diffuse interstitial fibrosis is consistently present, evidenced by significantly elevated ECV that is particularly pronounced in patients with diabetic complications (27). In a subset of individuals, focal non-ischemic scarring is also detectable as mid-wall LGE. These findings collectively define an early diabetic cardiomyopathy phenotype characterized by hypertrophy, impaired perfusion, diffuse fibrosis, and, in some cases, focal scarring—well before overt systolic dysfunction or clinical heart failure develop (47).

Markman et al. found that left atrial minimum volume and total, passive, and active emptying fractions correlate with CVD events in asymptomatic diabetics, independent of conventional risk factors and maximum left atrial volume (35). Liu et al. found that PDSR was more sensitive to subclinical myocardial dysfunction than PSSR in uncomplicated T2DM, and that decreased perfusion was associated with diastolic dysfunction (34). Yeo et al. reported a new index, peak early-to-late diastolic strain rate ratio (PEDSR:PLDSR) that was significantly reduced in T2DM, and correlated with echocardiographic E/A, reflecting both reduced early relaxation and increased atrial contraction (28). Dattani et al. demonstrated that right ventricular end-diastolic volume and cardiac output, but none of the left ventricular variables, were independently related to low aerobic capacity, underlining the significance of the right heart assessment (25). Walters et al. found that alternative measures of cardiopulmonary exercise testing (heart rate recovery, VO_2_ recovery, and VO_2_ at ventilatory threshold) were associated with left ventricular filling pressure, concentric remodeling, aortic stiffness and microvascular dysfunction, implying that they may be used to stratify risk stratification (20).

### 4.2 Preclinical and review evidence

Several recent narrative reviews and animal studies highlight that diabetes causes subtle myocardial alterations that can be present long before heart failure. Specific reviews of multimodality imaging, points out that echocardiography, nuclear techniques and CMR are essential to unmask early LV structural and functional changes in diabetic cardiomyopathy (48, 49). Adel and Chen suggest that chronic hyperglycemia and insulin resistance cause oxidative stress, inflammation, fibrosis and cardiomyocyte injury that culminate in concentric LV hypertrophy and impaired relaxation even in asymptomatic diabetes (48). These aberrations—diffuse fibrosis and altered energy metabolism—are measurable by advanced PET/MR imaging and echo biomarkers (50). These reviews validate our meta-analytic observations (increased diffuse fibrosis [higher ECV] and decreased myocardial strain/perfusion) and identify metabolic remodeling as the mechanism underpinning these imaging phenotypes.

A study by Korosoglou et al. used CMR and H-MRS to show that diastolic dysfunction in T2DM was more strongly associated with myocardial triglyceride content (myocardial steatosis) than with impaired perfusion reserve (51). This early discovery came even before they had the concept that vascular abnormalities alone might not be the most common cause of diabetic cardiomyopathy. Moreover, a large multi-center prognostic study by Giusca et al. was performed on more than 1,900 patients, involving the use of dobutamine stress CMR and LGE (52). It was shown that inducible ischemia is a strong predictor of adverse events in all patients but that myocardial scar is a strong prognostic marker in the diabetic sub-population. Our meta-analysis builds upon these previous findings by providing more robust pooled estimates for biomarkers such as ECV, MPRI, and LGE based on the most recent evidence, while firmly anchoring our results within this well-established context.

Bojer et al. demonstrated that microvascular dysfunction and diffuse fibrosis contribute independently to different components of diastolic dysfunction, with impaired myocardial perfusion primarily affecting early left ventricular relaxation (e.g., reduced e′ and elevated E/e′), while increased extracellular volume was more closely associated with later diastolic abnormalities, including altered filling dynamics and left atrial remodeling (53). Bojer et al. also found that early systolic dysfunction, especially decreased global longitudinal strain, is more significantly linked to microvascular dysfunction than fibrosis, indicating that the major contribution to the onset of early contractile dysfunction is the impaired myocardial perfusion (54). Moreover, two phenotypes of diabetic cardiomyopathy according to Madsen et al. were defined based on the data-driven cluster analysis, and they were different in ventricular geometry, hemodynamics, and myocardial perfusion reserve, which illustrates the heterogeneity of disease manifestation (47). All of this evidence suggests that CMR biomarkers are indicative of separate yet connected pathophysiological pathways that underlie both systolic and diastolic dysfunction and cannot be used as an early sign of subclinical disease.

Besides the main pieces of evidence that were incorporated in this review, there are other studies that further contextualize the role of CMR in diabetic cardiomyopathy. The association between diffuse fibrosis and systemic disease burden has also been supported by cross-sectional imaging studies that reveal expansion of the ECV is greater in patients with diabetic complications and is linked to established cardiovascular risk factors, including ischemic heart disease, autonomic neuropathy, and active smoking (27). Additionally, the circulating biomarkers in the form of fibroblast growth factor-23 have been linked to dysfunctional myocardial perfusion reserve and dysfunctional diastolics, which further support the interrelation between metabolic, vascular and myocardial changes (55). Lastly, previous systematic assessments of CMR methods show that CMR parameters like peak filling rates and left atrial performance are correlating with known diastolic dysfunction measures, which underscores the increased use of CMR as a complementary method of functional cardiac evaluation (56). Taken together, these results highlight the multifactorial and complicated character of myocardial involvement in diabetes and the need to incorporate CMR biomarkers into a larger pathophysiological context.

Overall, the evidence described above indicates that CMR biomarkers have the potential to demonstrate various aspects of diabetic involvement of the myocardium, such as vascular, interstitial, and functional changes. Nevertheless, these results are still tentative since the number of studies is low and the approaches are heterogeneous, with the majority of them being cross-sectional. Although multiparametric CMR has potential as a tool to detect subclinical disease, its use in early detection and risk stratification needs validation in larger well-designed prospective studies using standard imaging protocols.

### 4.3 Limitations and future direction

The limitations of this review were the limited number of eligible studies, the heterogeneity in imaging protocols, the predominantly cross-sectional study, and the generalizability of the findings. The standardization of CMR biomarkers (MPRI, ECV, and T1 mapping) was inconsistent, and most studies were conducted in type 2 diabetes, leaving a gap in knowledge for type 1 diabetes and other populations. The ROB was assessed as moderate to serious for some domains, particularly for selection and reporting bias. The other significant limitation is that it includes and synthesizes studies that have heterogenous designs and endpoints, including cross-sectional diagnostic comparisons as well as longitudinal and prognostic cohort studies. Such conceptual heterogeneity restricts the capacity to make conclusive findings on either diagnostic performance or prognostic value since they are different clinical questions.

Future studies should prioritize larger, prospective, multicenter studies with standardized imaging protocols and long-term follow-up to establish the prognostic value of these biomarkers. Combining CMR with other complementary imaging modalities and aligning preclinical studies with human protocols could improve the translational impact. Interventional trials using imaging biomarkers as surrogate endpoints are needed to evaluate their potential in guiding therapy and improving clinical outcomes in diabetes.

## 5 Conclusion

CVD is the most prominent cause of morbidity and mortality in patients with DM and subclinical myocardial involvement is a significant aspect of the disease progression. CMR biomarkers provide a noninvasive method to describe myocardial alterations such as fibrosis, microvascular dysfunction and structural remodeling. Although this systematic review and meta-analysis indicate a consistent tendency, including higher ECV, higher prevalence of LGE, and lower MPRI in diabetic groups, the results must be interpreted with caution because the number of studies is small, and their methodologies are not homogeneous. Additional studies also support the utility of CMR for detecting left atrial and right ventricular dysfunction, diastolic strain abnormalities, and impaired exercise capacity in patients with diabetes, while emphasizing the need for further methodological standardization. Altogether, the existing evidence indicates the perspective of multiparametric CMR as a viable tool, yet its clinical practice in early diagnosis and risk stratification is yet to be confirmed in large, prospective, and standardized studies.

## Data Availability

The original contributions presented in the study are included in the article/Supplementary Material, further inquiries can be directed to the corresponding author/s.
