# Supplementary material for: Cardiac magnetic resonance biomarkers for cardiovascular complications in diabetes: a systematic review and meta-analysis
Source: Front Cardiovasc Med. 2026 May 28;13:1817110. doi: 10.3389/fcvm.2026.1817110 (PMC13253232; doi:10.3389/fcvm.2026.1817110)
Supplement: Supplementary file 1 [file Table1.docx]

The core search syntax applied in PubMed/MEDLINE was as follows. The same syntax with Scopus, Embase, and ScienceDirect-specific requirements was utilized, with search in titles, abstracts and key words.

(

"Diabetes Mellitus"[Mesh]

OR "diabetes mellitus"[tiab]

OR "type 1 diabetes"[tiab]

OR "type 2 diabetes"[tiab]

OR T1DM[tiab]

OR T2DM[tiab]

OR "prediabetes"[tiab]

)

AND

(

"Magnetic Resonance Imaging"[Mesh]

OR "cardiac magnetic resonance"[tiab]

OR "cardiac MRI"[tiab]

OR CMR[tiab]

OR "cardiovascular magnetic resonance"[tiab]

)

AND

(

"T1 mapping"[tiab]

OR "T2 mapping"[tiab]

OR "T2* mapping"[tiab]

OR "extracellular volume"[tiab]

OR ECV[tiab]

OR "late gadolinium enhancement"[tiab]

OR LGE[tiab]

OR "myocardial strain"[tiab]

OR strain[tiab]

OR "myocardial perfusion"[tiab]

OR perfusion[tiab]

OR "perfusion reserve"[tiab]

OR MPRI[tiab]

OR "fat fraction"[tiab]

OR "parametric mapping"[tiab]

)

AND

(

"Cardiovascular Diseases"[Mesh]

OR "cardiovascular disease"[tiab]

OR cardiomyopathy[tiab]

OR "myocardial fibrosis"[tiab]

OR ischemia[tiab]

OR "microvascular dysfunction"[tiab]

OR "heart failure"[tiab]

)

AND

(

"2015/01/01"[Date - Publication] : "2025/09/30"[Date - Publication]

)

AND

(

english[lang]

)
